# 2,6-Dichloro­benzaldehyde oxime

**DOI:** 10.1107/S1600536808033217

**Published:** 2008-10-18

**Authors:** Feng-Yu Bao

**Affiliations:** a Department of Applied Chemistry, College of Science, Henan Agricultural University, Zhengzhou 450002, People’s Republic of China

## Abstract

In the title compound, C_7_H_5_Cl_2_NO, there are two mol­ecules in the asymmetric unit. The mol­ecules are essentially identical. Each mol­ecule is connected to a symmetry-related mol­ecule through an inversion center by O—H⋯N hydrogen bonds, building an *R*_2_^2^(6) graph-set motif.

## Related literature

For related literature, see: Xu & Jin (1999 ▶). For graph-set notation, see: Etter *et al.* (1990 ▶); Bernstein *et al.* (1995 ▶).
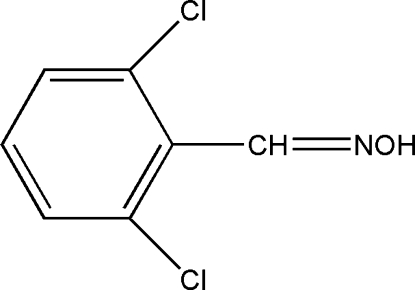


## Experimental

### 

#### Crystal data

C_7_H_5_Cl_2_NO
*M* *_r_* = 190.02
Triclinic, 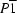

*a* = 3.8074 (1) Å
*b* = 14.3712 (2) Å
*c* = 14.3835 (3) Å
α = 89.108 (1)°
β = 88.545 (1)°
γ = 85.296 (1)°
*V* = 784.04 (3) Å^3^
*Z* = 4
Mo *K*α radiation
μ = 0.76 mm^−1^
*T* = 296 (2) K
0.26 × 0.24 × 0.16 mm

#### Data collection

Bruker SMART CCD area-detector diffractometer
Absorption correction: multi-scan (*SADABS*; Bruker, 1998 ▶) *T*_min_ = 0.818, *T*_max_ = 0.884
11107 measured reflections
3235 independent reflections
2809 reflections with *I* > 2σ(*I*)
*R*_int_ = 0.018

#### Refinement

*R*[*F* ^2^ > 2σ(*F* ^2^)] = 0.032
*wR*(*F* ^2^) = 0.085
*S* = 1.06
3235 reflections
201 parameters
H-atom parameters constrained
Δρ_max_ = 0.40 e Å^−3^
Δρ_min_ = −0.31 e Å^−3^

### 

Data collection: *SMART* (Bruker, 1998 ▶); cell refinement: *SAINT* (Bruker, 1998 ▶); data reduction: *SAINT*; program(s) used to solve structure: *SHELXS97* (Sheldrick, 2008 ▶); program(s) used to refine structure: *SHELXL97* (Sheldrick, 2008 ▶); molecular graphics: *ORTEPIII* (Burnett & Johnson, 1996 ▶), *ORTEP-3 for Windows* (Farrugia, 1997 ▶) and *PLATON* (Spek, 2003 ▶); software used to prepare material for publication: *SHELXL97*.

## Supplementary Material

Crystal structure: contains datablocks global, I. DOI: 10.1107/S1600536808033217/dn2391sup1.cif

Structure factors: contains datablocks I. DOI: 10.1107/S1600536808033217/dn2391Isup2.hkl

Additional supplementary materials:  crystallographic information; 3D view; checkCIF report

## Figures and Tables

**Table 1:** Hydrogen-bond geometry (Å, °)

*D*—H⋯*A*	*D*—H	H⋯*A*	*D*⋯*A*	*D*—H⋯*A*
O1—H1⋯N1^i^	0.82	2.14	2.854 (2)	145
O2—H2⋯N2^ii^	0.82	2.15	2.850 (2)	144

Symmetry codes: (i) 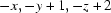; (ii) 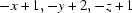.

**Fig. 1.**
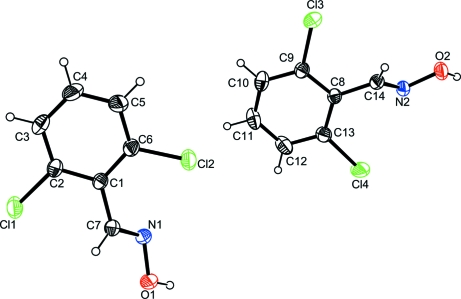
The asymmetric unit of (I) with the atom-labelling scheme. Displacement ellipsoids are drawn at the 30% probability level. H atoms are represented as small spheres of arbitrary radii.

**Fig. 2.**
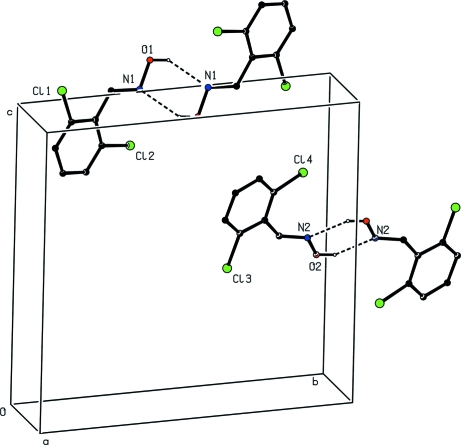
Partial packing view of compound ( I ), showing the formation of dimer through R22(6) graph set motif. H bonds are represented as dashed lines. H atoms not involved in hydrogen bonding have been omitted. [Symmetry codes: (i) -x, -y+1, -z+2; (ii) -x+1 , -y+2, -z+1.]

## supplementary crystallographic information

### Comment

2,6-Dichlorobenzaldehyde oxime, is an important intermediate for organic synthesis(Xu & Jin,1999). As part of our task, we have synthesized the title compound (I) .

In the title compound, C_7_H_6_Cl_2_NO, there are two molecules in the asymmetric unit. Both molecules are roughly identical, the oxime fragment is twisted with respect to the dichlorobenzene ring by 53.83 (11)° and 42.99 (14)° respectively (Fig. 1).

Each molecule is connected to its symmetry related one through inversion center by O-H···N hydrogen bonds building a *R*_2_^2^(6) graph-set motif (Etter *et al.*, 1990; Bernstein *et al.*, 1995) (Fig. 2 and Table 1).

### Experimental

2,6-dichlorobenzaldehyde (1 mmol) was dissolved in anhydrous methanol, hydroxylamine hydrochloride and sodium carbonate were added to this, the mixture was stirred for 3 h at room temperature. The product was isolated and recrystallized in dichloromethane, colourless single crystals of (I) was obtained after 5 d.

### Refinement

All H atoms were placed in calculated position and treated as riding on their parent atoms with C—H=0.93Å or O—H=0.82 Å with *U*_iso_(H)=1.2*U*_eq_(C) or 1.5*U*_eq_(O) for the hydroxyl H atom.

### Crystal data

**Table:**

C_7_H_5_Cl_2_NO	*Z* = 4
*M**_r_* = 190.02	*F*(000) = 384
Triclinic, *P*1	*D*_x_ = 1.610 Mg m^−^^3^
Hall symbol: -P 1	Mo *K*α radiation, λ = 0.71073 Å
*a* = 3.8074 (1) Å	Cell parameters from 5529 reflections
*b* = 14.3712 (2) Å	θ = 2.8–27.4°
*c* = 14.3835 (3) Å	µ = 0.76 mm^−^^1^
α = 89.108 (1)°	*T* = 296 K
β = 88.545 (1)°	Block, colourless
γ = 85.296 (1)°	0.26 × 0.24 × 0.16 mm
*V* = 784.04 (3) Å^3^	

### Data collection

**Table:**

Bruker SMART CCD area-detector diffractometer	3235 independent reflections
Radiation source: fine-focus sealed tube	2809 reflections with *I* > 2σ(*I*)
graphite	*R*_int_ = 0.018
ω scans	θ_max_ = 26.5°, θ_min_ = 1.4°
Absorption correction: multi-scan (SADABS; Bruker, 1998)	*h* = −4→4
*T*_min_ = 0.818, *T*_max_ = 0.884	*k* = −17→17
11107 measured reflections	*l* = −18→18

### Refinement

**Table:**

Refinement on *F*^2^	Primary atom site location: structure-invariant direct methods
Least-squares matrix: full	Secondary atom site location: difference Fourier map
*R*[*F*^2^ > 2σ(*F*^2^)] = 0.032	Hydrogen site location: inferred from neighbouring sites
*wR*(*F*^2^) = 0.085	H-atom parameters constrained
*S* = 1.06	*w* = 1/[σ^2^(*F*_o_^2^) + (0.0352*P*)^2^ + 0.3155*P*] where *P* = (*F*_o_^2^ + 2*F*_c_^2^)/3
3235 reflections	(Δ/σ)_max_ = 0.001
201 parameters	Δρ_max_ = 0.40 e Å^−^^3^
0 restraints	Δρ_min_ = −0.31 e Å^−^^3^

### Special details

**Table:**

Geometry. All esds (except the esd in the dihedral angle between two l.s. planes) are estimated using the full covariance matrix. The cell esds are taken into account individually in the estimation of esds in distances, angles and torsion angles; correlations between esds in cell parameters are only used when they are defined by crystal symmetry. An approximate (isotropic) treatment of cell esds is used for estimating esds involving l.s. planes.
Refinement. Refinement of *F*^2^ against ALL reflections. The weighted *R*-factor *wR* and goodness of fit *S* are based on *F*^2^, conventional *R*-factors *R* are based on *F*, with *F* set to zero for negative *F*^2^. The threshold expression of *F*^2^ > σ(*F*^2^) is used only for calculating *R*-factors(gt) *etc*. and is not relevant to the choice of reflections for refinement. *R*-factors based on *F*^2^ are statistically about twice as large as those based on *F*, and *R*- factors based on ALL data will be even larger.

### Fractional atomic coordinates and isotropic or equivalent isotropic displacement parameters (Å^2^)

**Table:**

	*x*	*y*	*z*	*U*_iso_*/*U*_eq_	
Cl1	0.16981 (16)	0.12516 (3)	1.04669 (4)	0.06328 (16)	
Cl2	−0.28140 (15)	0.39071 (3)	0.79443 (4)	0.05813 (16)	
N1	−0.0849 (4)	0.39836 (10)	0.99884 (10)	0.0430 (3)	
O1	−0.2185 (4)	0.44211 (9)	1.07941 (9)	0.0574 (4)	
H1	−0.1779	0.4973	1.0774	0.086*	
C1	−0.0441 (4)	0.25470 (11)	0.91699 (11)	0.0371 (4)	
C2	0.1056 (5)	0.16371 (12)	0.93282 (13)	0.0426 (4)	
C3	0.2132 (6)	0.10360 (13)	0.86200 (16)	0.0551 (5)	
H3	0.3128	0.0437	0.8752	0.066*	
C4	0.1703 (7)	0.13390 (15)	0.77145 (16)	0.0653 (6)	
H4	0.2431	0.0943	0.7229	0.078*	
C5	0.0202 (6)	0.22243 (15)	0.75209 (14)	0.0599 (5)	
H5	−0.0105	0.2422	0.6908	0.072*	
C6	−0.0839 (5)	0.28136 (12)	0.82397 (13)	0.0434 (4)	
C7	−0.1573 (5)	0.31429 (12)	0.99624 (12)	0.0416 (4)	
H7	−0.2847	0.2895	1.0454	0.050*	
Cl3	0.60840 (16)	0.62545 (3)	0.44764 (4)	0.06139 (16)	
Cl4	0.35612 (19)	0.88605 (4)	0.71520 (4)	0.07117 (19)	
N2	0.4237 (4)	0.89871 (10)	0.50330 (10)	0.0429 (3)	
O2	0.2629 (4)	0.94385 (9)	0.42708 (9)	0.0538 (3)	
H2	0.3079	0.9987	0.4256	0.081*	
C8	0.5024 (4)	0.75283 (11)	0.58413 (11)	0.0384 (4)	
C9	0.6303 (5)	0.66160 (12)	0.56212 (13)	0.0421 (4)	
C10	0.7785 (5)	0.59945 (13)	0.62661 (16)	0.0546 (5)	
H10	0.8605	0.5393	0.6093	0.065*	
C11	0.8035 (6)	0.62748 (15)	0.71651 (16)	0.0618 (6)	
H11	0.9070	0.5864	0.7604	0.074*	
C12	0.6773 (6)	0.71567 (15)	0.74256 (14)	0.0612 (6)	
H12	0.6931	0.7340	0.8039	0.073*	
C13	0.5265 (5)	0.77725 (12)	0.67727 (13)	0.0477 (4)	
C14	0.3507 (5)	0.81491 (11)	0.51094 (12)	0.0406 (4)	
H14	0.1973	0.7919	0.4693	0.049*	

### Atomic displacement parameters (Å^2^)

**Table:**

	*U*^11^	*U*^22^	*U*^33^	*U*^12^	*U*^13^	*U*^23^
Cl1	0.0848 (4)	0.0441 (3)	0.0602 (3)	0.0013 (2)	−0.0151 (3)	0.0096 (2)
Cl2	0.0817 (4)	0.0398 (3)	0.0525 (3)	0.0009 (2)	−0.0155 (2)	0.0047 (2)
N1	0.0565 (9)	0.0348 (7)	0.0375 (8)	−0.0012 (6)	−0.0006 (6)	−0.0057 (6)
O1	0.0891 (11)	0.0393 (7)	0.0429 (7)	−0.0001 (7)	0.0089 (7)	−0.0099 (6)
C1	0.0395 (9)	0.0296 (8)	0.0428 (9)	−0.0062 (6)	−0.0018 (7)	−0.0032 (7)
C2	0.0440 (10)	0.0329 (8)	0.0514 (10)	−0.0052 (7)	−0.0040 (8)	−0.0010 (7)
C3	0.0587 (12)	0.0330 (9)	0.0729 (14)	0.0009 (8)	0.0011 (10)	−0.0095 (9)
C4	0.0859 (16)	0.0477 (12)	0.0617 (13)	−0.0014 (11)	0.0098 (12)	−0.0206 (10)
C5	0.0861 (16)	0.0497 (11)	0.0445 (11)	−0.0080 (10)	0.0018 (10)	−0.0081 (9)
C6	0.0516 (10)	0.0331 (8)	0.0462 (10)	−0.0065 (7)	−0.0038 (8)	−0.0026 (7)
C7	0.0480 (10)	0.0349 (9)	0.0417 (9)	−0.0035 (7)	0.0023 (7)	0.0002 (7)
Cl3	0.0811 (4)	0.0450 (3)	0.0574 (3)	0.0006 (2)	0.0000 (3)	−0.0139 (2)
Cl4	0.1140 (5)	0.0476 (3)	0.0511 (3)	−0.0031 (3)	0.0111 (3)	−0.0128 (2)
N2	0.0526 (9)	0.0351 (7)	0.0403 (8)	−0.0002 (6)	−0.0031 (6)	0.0031 (6)
O2	0.0744 (9)	0.0380 (7)	0.0486 (7)	0.0001 (6)	−0.0127 (7)	0.0071 (6)
C8	0.0419 (9)	0.0317 (8)	0.0422 (9)	−0.0069 (7)	0.0001 (7)	0.0022 (7)
C9	0.0444 (10)	0.0337 (8)	0.0488 (10)	−0.0068 (7)	−0.0008 (8)	−0.0002 (7)
C10	0.0569 (12)	0.0343 (9)	0.0728 (14)	−0.0048 (8)	−0.0082 (10)	0.0080 (9)
C11	0.0744 (15)	0.0481 (11)	0.0644 (13)	−0.0124 (10)	−0.0206 (11)	0.0208 (10)
C12	0.0871 (16)	0.0552 (12)	0.0443 (11)	−0.0219 (11)	−0.0113 (10)	0.0082 (9)
C13	0.0630 (12)	0.0371 (9)	0.0441 (10)	−0.0105 (8)	0.0017 (8)	−0.0001 (7)
C14	0.0451 (10)	0.0346 (9)	0.0422 (9)	−0.0027 (7)	−0.0020 (7)	−0.0020 (7)

### Geometric parameters (Å, °)

**Table:**

Cl1—C2	1.7376 (19)	Cl3—C9	1.7403 (19)
Cl2—C6	1.7370 (18)	Cl4—C13	1.7336 (19)
N1—C7	1.262 (2)	N2—C14	1.261 (2)
N1—O1	1.3919 (19)	N2—O2	1.3945 (18)
O1—H1	0.8200	O2—H2	0.8200
C1—C6	1.394 (2)	C8—C13	1.397 (2)
C1—C2	1.401 (2)	C8—C9	1.399 (2)
C1—C7	1.471 (2)	C8—C14	1.468 (2)
C2—C3	1.378 (3)	C9—C10	1.377 (3)
C3—C4	1.376 (3)	C10—C11	1.368 (3)
C3—H3	0.9300	C10—H10	0.9300
C4—C5	1.379 (3)	C11—C12	1.372 (3)
C4—H4	0.9300	C11—H11	0.9300
C5—C6	1.376 (3)	C12—C13	1.383 (3)
C5—H5	0.9300	C12—H12	0.9300
C7—H7	0.9300	C14—H14	0.9300
			
C7—N1—O1	111.93 (15)	C14—N2—O2	111.98 (15)
N1—O1—H1	109.5	N2—O2—H2	109.5
C6—C1—C2	115.78 (16)	C13—C8—C9	115.73 (16)
C6—C1—C7	124.33 (15)	C13—C8—C14	124.76 (16)
C2—C1—C7	119.86 (15)	C9—C8—C14	119.50 (15)
C3—C2—C1	123.02 (18)	C10—C9—C8	122.98 (18)
C3—C2—Cl1	118.10 (15)	C10—C9—Cl3	118.38 (15)
C1—C2—Cl1	118.86 (14)	C8—C9—Cl3	118.63 (14)
C4—C3—C2	118.71 (18)	C11—C10—C9	119.02 (19)
C4—C3—H3	120.6	C11—C10—H10	120.5
C2—C3—H3	120.6	C9—C10—H10	120.5
C3—C4—C5	120.56 (19)	C10—C11—C12	120.62 (19)
C3—C4—H4	119.7	C10—C11—H11	119.7
C5—C4—H4	119.7	C12—C11—H11	119.7
C6—C5—C4	119.7 (2)	C11—C12—C13	119.8 (2)
C6—C5—H5	120.2	C11—C12—H12	120.1
C4—C5—H5	120.2	C13—C12—H12	120.1
C5—C6—C1	122.23 (17)	C12—C13—C8	121.80 (18)
C5—C6—Cl2	117.14 (15)	C12—C13—Cl4	117.71 (16)
C1—C6—Cl2	120.61 (13)	C8—C13—Cl4	120.46 (14)
N1—C7—C1	121.30 (16)	N2—C14—C8	121.45 (16)
N1—C7—H7	119.4	N2—C14—H14	119.3
C1—C7—H7	119.4	C8—C14—H14	119.3

### Hydrogen-bond geometry (Å, °)

**Table:**

*D*—H···*A*	*D*—H	H···*A*	*D*···*A*	*D*—H···*A*
O1—H1···N1^i^	0.82	2.14	2.854 (2)	145
O2—H2···N2^ii^	0.82	2.15	2.850 (2)	144

Symmetry codes: (i) −*x*, −*y*+1, −*z*+2; (ii) −*x*+1, −*y*+2, −*z*+1.
